# Synthesis and Local Characterization of CoO Nanoparticles in Distinct Phases: Unveiling Polymorphic Structures

**DOI:** 10.1021/acsomega.4c05308

**Published:** 2024-10-10

**Authors:** Suzilene V. Santos, Cleidilane S. Costa, Waldeci Paraguassu, Crystian W. C. Silva, Larissa Otubo, Katiusse S. Souza, Bruno S. Correa, Arnaldo A. Miranda-Filho, Wanderson L. Ferreira, Artur W. Carbonari, Gabriel A. Cabrera-Pasca

**Affiliations:** † Programa de Pós-Graduação em Ciência e Engenharia de Materiais − PPGCEM, Universidade Federal do Pará (UFPA), Ananindeua, Pará 67130-660, Brazil; ‡ Faculdade de Ciências Exatas e Tecnologia, Universidade Federal do Pará (UFPA), Abaetetuba, Pará 684440-000, Brazil; § Instituto de Pesquisas Energéticas e Nucleares IPEN-CNEN/SP, São Paulo, São Paulo 05508-000, Brazil; ∥ EP Department, European Organization for Nuclear Research (CERN), Geneva CH-1211, Switzerland

## Abstract

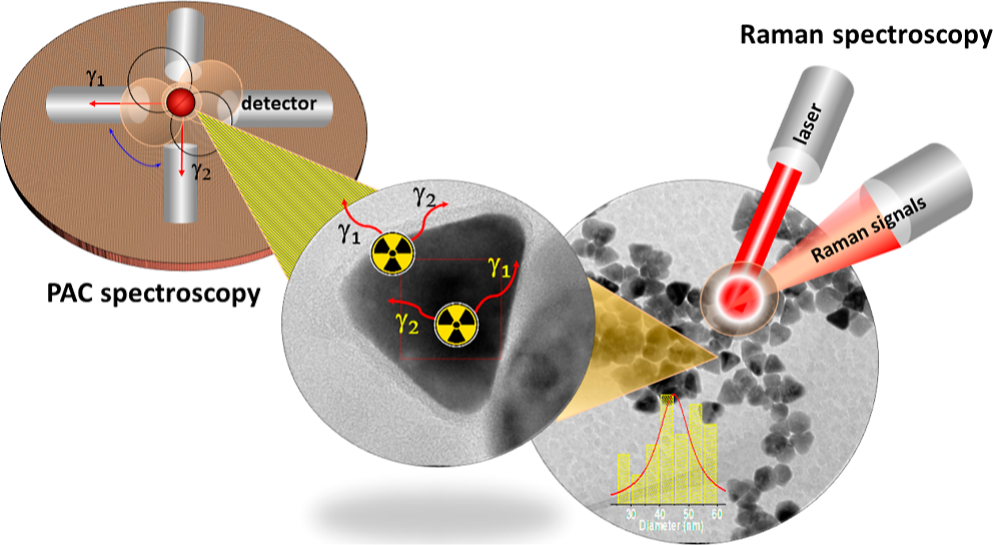

The advancement of functional nanomaterials has become a major focus of recent research, driven by the exceptional properties these materials display compared to their macroscopic (bulk) counterparts. Cobalt oxide nanoparticles (CoO-NPs) stand out primarily for their catalytic and magnetic properties, which can enable a range of technological applications, such as advanced catalysts, drug delivery systems, implants, prosthetics, sensors. However, in addition to the dependence on factors such as size, morphology, and functionalization, the properties of CoO-NPs are significantly influenced by the crystal structure. Therefore, local investigation into the polymorphic structures of CoO at the nanometric scale may provide new insights into the local structural and magnetic characteristics of these systems. In this report, we address the synthesis and local characterization of cobalt oxide (CoO) nanoparticles in the rock-salt cubic fcc-CoO and Wurtzite hpc-CoO phases, obtained through thermal decomposition. We analyze the influence of oleylamine and oleic acid ligands on the structural and morphological control of these systems. The obtained nanoparticles were characterized using conventional techniques such as X-ray diffraction (XRD), transmission electron microscopy, Raman spectroscopy, and Fourier-transform infrared spectroscopy. Local characterization was carried out by the perturbed angular correlation (PAC) nuclear technique using the radioactive tracer ^111^In(^111^Cd). Measurements were conducted at 295 and 10 K to investigate possible magnetic phase transitions in these systems. XRD results confirmed the formation of fcc-CoO and hcp-CoO phases. The phase fcc was obtained with the pair of oleylamine and oleic acid ligands, while the phase hcp phase was synthesized using only oleylamine. Additionally, nanoparticles synthesized with oleylamine and oleic acid exhibited better morphological control compared to those produced with only oleylamine. Raman spectroscopy analyses suggest a phase transformation process resulting in Co_3_O_4_. PAC results for hyperfine interactions at the ^111^In(^111^Cd) probe nucleus, indicate that the hcp-CoO phase shows smaller hyperfine magnetic interactions (*B*_hf_ = 1 T) compared to the fcc-CoO phase (*B*_hf_ = 17 T). This suggests the mechanism of superexchange interactions, which are strongly influenced by the Co–O–Co bond angle, which is 110*°* for the hpc-CoO phase and 180*°* for the fcc-CoO phase due to the geometries of the systems.

## Introduction

In recent years, an enormous amount of effort has been focused on developing procedures for the synthesis of nanostructured materials. Among all of these methods, the bottom-up synthesis strategy has received substantial attention from the scientific community because of its ability to allow controlled synthesis of nanomaterials. In contrast to bulk-type macrometric objects, this technique allows for exact control over many characteristics such as size, shape, composition, and structural order, among others.^1,2^ All of which have a direct impact on the intrinsic physicochemical characteristics of nanometric objects. Nanoparticle (NPs) science and related technologies have enabled important advances in a variety of areas, including nanomedicine, engineering, and agriculture, among others. These technological advances have led the way to the development of novel sensors, actuators, carriers, and other devices, resulting in the creation of novel drugs, ultraresponsive diagnostics, implants, and prostheses, as well as improved drug delivery systems and vaccines.^3,4^

At the nanoscale, a substantial portion of the atoms present in the system are on or near the surface, resulting in intriguing processes that are currently being studied extensively. For example, the origin of ferromagnetic behavior in nanoscale materials, which typically exhibit paramagnetic behavior, remains an open question. Some researchers believe this unusual magnetism is due to surface effects and that bulk defects (such as vacancies) can cause uncompensated spins, resulting in a ferromagnetic response.^4,5^ Another topic of research in nanoscale systems is the stability of crystalline phases, which are rarely stable at larger sizes, resulting in new chemical–physical properties such as ferromagnetic-like responses.^6,7^

Particularly, cobalt oxide nanoparticles (Co-NPs), including rock-salt-CoO, Wurtzite-CoO, zincblend-CoO, and cobaltite (Co_3_O_4_), which corresponds to the cubic spinel phase of the Co oxide, have sparked significant interest due to their widespread applications in a variety of industrial sectors, including rechargeable lithium batteries,^8^ catalysts,^9^gas sensors,^10^ and biomedicine.^11^ This broad range of applications is attributed to the different magnetic, mechanical, optical, chemical, and electrical properties exhibited by the crystallo- graphic phases of cobalt oxide when reduced to the nanoscale.^12^

The polymorphism of the CoO-NPs, resulting in different crystalline structures, depends on the synthesis route and the reduction atmosphere, owing to the different oxidation states of cobalt. Particularly, the fcc-CoO rocksalt structure remains thermodynamically stable, while the hcp-CoO structure is relatively unstable and can be easily transformed into a cubic structure by application of heat and pressure.^13^

The development of functionalized CoO-NPs with well-defined structure and morphology is of particular interest for various applications. For instance, in catalytic applications (such as oxidation, hydrogenation, and hydroamination reactions), the functionalization of the nanoparticle surface is crucial. Specifically, this occurs because the adsorbed ligands on the surface act as mediators, facilitating the interaction between the nanoparticle and its surroundings. This characteristic enables the manipulation of not only the hydrophilicity of the nanoparticles, the thermal stability, and mechanical resistance but also their electronic properties. This interaction can influence the electronic properties not only of the direct interaction sites (ligand-surface), but also of adjacent sites, allowing fine-tuning of properties related to the activity and selectivity of the catalyst.^14^ Another interesting aspect is the role of surface functionalization in governing the morphology of CoO nanostructures and its subsequent impact on the applications of these systems. For example, Cai et al. successfully synthesized interconnected 2D CoO nanosheets, which, after thermal treatment, were transformed into Co_3_O_4_ while maintaining their original morphology. From this perspective, the significant catalytic activity of the Co_3_O_4_ spinel phase, coupled with the 2D morphology of the system, enabled the presence of numerous active sites of Co^3+^and O_2_ on the surface. This configuration contributed to the material’s exceptional catalytic performance.^15^ Thus, the customization of these fundamental aspects for CoO nanoparticles can be adjusted using chemical protocols by varying parameters such as precursor concentration, reaction temperature, reaction time, and the type of ligands used, with oleic acid and oleylamine being frequently reported in the literature.^16,17^

For example, according to previous studies realized by He et al., hpc-CoO NPs can be obtained by the thermal decomposition method at 220 ^*°*^C for 1 h, using cobalt salts and oleylamine, as a reducer and surfactant.^18^ Ravindra et al. stated that hpc-CoO NPs were formed by thermolysis at 296 ^*°*^C, under mechanical stirring.^13^ Meanwhile, Deori and Deka reported that fcc-CoO NPs were created by solvothermal-driven chemical processes at a temperature of 220 ^*°*^C.^19^ some studies are available in the literature, aspects related to the formation mechanism, as well as the physical and chemical properties of hpc-CoO nanoparticles, remain challenging and largely unexplored due to their phase instability.

Therefore, a localized study of these systems using experimental nuclear techniques is crucial as they provide detailed information at the atomic scale in nanoparticles. This local information can be correlated with results obtained through other techniques such as X-ray diffraction, Raman spectroscopy, and transmission electron microscopy (TEM). This allows for a comprehensive understanding of the systems. This set of information allows for excellent characterization in terms of crystalline structure, stoichiometry, and crystalline and magnetic phase transitions that occur in the vicinity of surfaces or interfaces of the nanomaterials.^20,21^

Among nuclear techniques, nuclear magnetic resonance (NMR), Mössbauer spectroscopy, and perturbed angular correlation (PAC) stand out. The PAC technique is particularly notable as a nonresonant nuclear method, making it especially useful for local structural and magnetic characterization over a wide range of temperatures^22,23^ This technique measures the angular distribution of gamma photons emitted by a radioactive probe nucleus, allowing for detailed local characterization of the material properties. The precision of this spectroscopic technique arises from the hyperfine interactions between suitable probe nuclei and their near environments into samples. These interactions provide valuable information on the local electric field and the magnetic environment surrounding the probe, which contributes to a deeper understanding of the material characteristics.^24^

The angular distribution pattern extracted from a PAC experiment is usually expressed in terms of hyperfine parameters, such as the magnetic interaction frequency (ν_M_), the electric quadrupole frequency (ν_Q_) and the asymmetry parameter (η). Since the PAC technique needs the presence of radioactive atoms inside the nanoparticle which can be difficult to produce through implantation processes, its application in the re- search of nanoparticles is still relatively unexplored. However, if the radionuclides utilized in the PAC technique are added during the nucleation and growth processes of nanomaterials, this limitation can be circumvented. This allows for atomic-level investigation of several particle regions, including the coating, interface, and core of the nanoparticles. For example, the PAC technique has been applied recently to further understand the size influence on magnetism in CoO-NPs^25^ and to investigate the impact of ligand pairs in the synthesis and stabilization of Fe_3_O_4_ nanoparticles.^21^ Furthermore, because PAC is a local method, it allows the mapping of vacancies and crystal structural distortions surrounding a specific site occupied by probe.^25,26^

In this report, we address the synthesis and local characterization of cobalt oxide (CoO) nanoparticles in the fcc and hcp phases, obtained through thermal decomposition. To obtain nanoparticles with precise dimensions and morphology, we rigorously controlled the process parameters, including the choice of chemicals, nucleation temperature, growth time, and reaction time. For local characterization, we used the PAC technique using the radioactive tracer ^111^In(^111^Cd), with measurements conducted at 295 and 10 K to investigate possible magnetic phase transitions in these systems. In addition, we used Raman spectroscopy to assess the structural stability of the nanoparticles. The data obtained from these spectroscopic techniques were correlated with structural and morphological information obtained by TEM (TEM/HRTEM), X-ray diffraction (XRD), and Fourier-transform infrared spectroscopy (FTIR). This integrated approach enabled a detailed understanding of the nanoparticle properties and their correlation with the observed physical characteristics.

## Experimental Section

### Synthesis of Fcc-CoO and Hcp-CoO

Cobalt(III) acetylacetonate (Co(acac)_3_) was used as the metal precursor in the synthesis of cobalt oxide nanoparticles, together with varying quantities of oleylamine (C_18_H_37_N) and oleic acid (C_18_H_34_O_2_). The reagents were placed into a flask with three necks as part of the synthesis process. To promote heat exchange between the equipment and the container, the reaction flask was set atop a heating mantle that was covered with aluminum foil. To monitor the internal temperature of the flask while it was being heated, a thermocouple was placed inside. Furthermore, a cold water flow was utilized in an Allihn condenser to avoid stoichiometric losses during the synthesis. Additionally, a connection for the flow of nitrogen was included, which provided an inert atmosphere for the synthesis.

Carefully controlled experimental conditions were employed to ensure the synthesis of cobalt oxide nanoparticles with the intended size and shape. Oleic acid and oleylamine may have a significant impact on crystal orientation, size control, and nanoparticle stabilization. The samples underwent a variety of steps and conditions during the synthesis process. Following a heating stage at a rate of 6 ^*°*^C per minute until reaching the nucleation temperature of 130 ^*°*^C for 10 min, the temperature was increased to certain values for each synthesized sample and held for defined reaction times. For a sample named S_1_, oleylamine, oleic acid, and Co(acac)_3_ were used as reagents. Sample S_1_ underwent a reaction lasting 1 h at an average temperature of 270 ^*°*^C. Similarly, the other sample, named S_2_, was synthesized by reacting only oleylamine and Co(acac)_3_ for 3 h at 220 ^*°*^C.

After synthesis, the resulting solution was cooled and dissolved in various solvents, such as ethyl alcohol, toluene, and methyl ethyl ketone. These solvents were carefully selected to aid in the mixing and dissolution of the compounds present in the sample. To ensure complete mixing, the solution was then cleaned with an ultrasonic device. The nanoparticles were then separated from the solvent and any other potential contaminants using centrifugation. Samples synthesized with each solvent were subjected to centrifugation for 20 min at a speed of 10,000 rpm. Finally, the samples were vacuum-dried using a desiccator and a vacuum pump, reaching a pressure of up to 10^–3^ bar.

After drying, a pure powder was obtained and the characteristics of the samples were analyzed. (1) For both measurements, TEM and high-resolution transmission electron microscopy (HRTEM), samples were prepared by depositing a portion of a nanoparticle suspension solution in isopropanol alcohol onto a grid coated with Formvar and carbon. The powdered samples were prepared by dispersing them in isopropanol in an ultrasonic bath, then pouring them onto a copper grid covered with collodion film, and allowing them to air-dry. The Jeol-JEM 2100 transmission electron microscope, operating at an acceleration voltage of 200 kV and available at the Laboratory of Microscopy and Microanalysis (LMM) of the Center for Science and Technology of Materials at the Nuclear and Energy Research Institute (CECTM/IPEN), was used to measure the size distribution and morphology of samples S_1_ and S_2_. The images were captured at various magnifications to assess the shape and size distribution of the sample.

(2) The crystalline structure was determined using XRD performed with a Rigaku SmarLab model equipped with a high-resolution 2D HPAD detector located at the Research Reactor Center (CERPq/IPEN). Cu Kα radiation (λ = 1.54056 Å) was used to collect data in an angular range of 20–80*°* at 40 kV and 40 mA, with a step size of 0.01*°*.

(3) Fourier transform infrared spectroscopy (FTIR) measurements were taken after the synthesis of the fcc-CoO and hcp -CoO to characterize the coating materials that involve them. The oleic acid and oleylamine included in the coating layer of the NPs can be identified through FTIR measurements. Infrared spectra were obtained using the Bruker VERTEX 70v spectrometer with a spectral resolution of 4 cm^–1^ from the UFPA high pressure and vibrational spectroscopy laboratory. Measurements were carried out between 500 and 4000 cm^–1^ with attenuated total reflectance (ATR). Additionally, (4) the Micro Raman spectrometer (Jobin Yvon-T64000) was used at the UFPA vibrational spectroscopy and high pressure laboratory to analyze the samples employing Raman spectroscopy. Using the Fityk fitting tool, the Lorentzian function was used to fit the active Raman modes.

For atomic scale characterization, (5) PAC spectroscopy experiments were carried out in the Laboratory of Hyperfine Interactions at CERPq/IPEN in São Paulo, Brazil, using a spectrometer equipped with four BaF_2_ detectors. In the preparation of the NP samples for the PAC measurements, specifically, 5 μL of radioactive InCl_3_ with an activity of 20 μCi was added during synthesis. The highest number of impurity atoms estimated from this action is around 2.5 × 10^11^. The precise concentration, however, is dependent on the diffusion of probe nuclei inside the sample and, consequently, on variables including reaction time, temperature, and ligand type. As a result, the final concentration cannot be measured; nonetheless, the estimate is approximately 100 ppb, which is insignificant enough to affect the sample characteristics.

**Figure 1:**
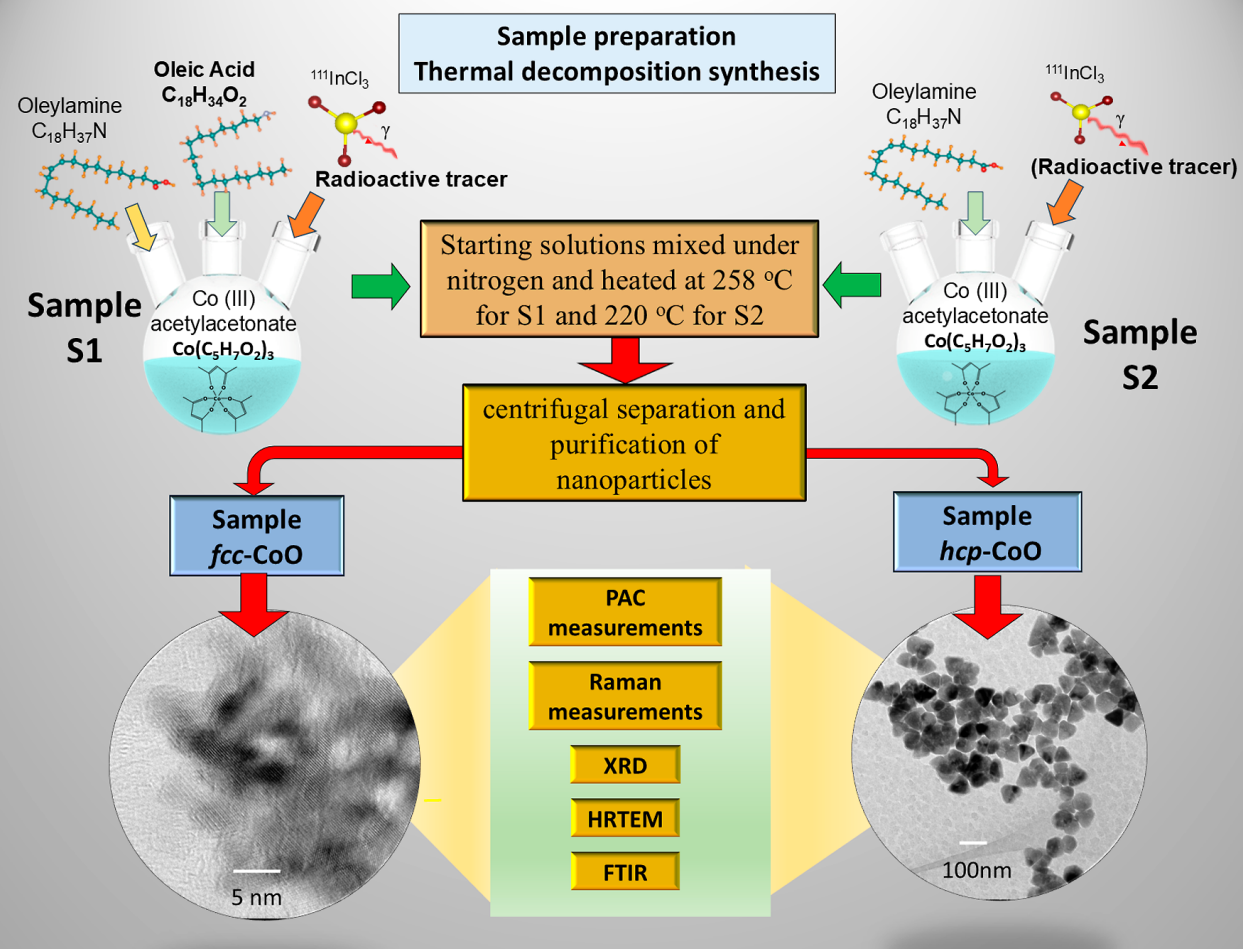
Schematic illustration showing the steps involved in the preparation and characterization of fcc-CoO (S_1_) and hcp-CoO (S_2_) nanoparticles, highlighting the synthesis conditions and reagents used. In the context of characterization techniques, local PAC analysis and Raman spectroscopy are emphasized, along with other techniques used such as XRD, HRTEM, and FTIR.

All these characterizations along with the synthesis of samples are shown schematically in Figure 1.

PAC spectroscopy is based on the observation of hyperfine interactions between nuclear moments and extra-nuclear magnetic fields (*B*_hf_) or with an electric field gradient (EFG). Details on the PAC measures and a description of the methodology can be found elsewhere.^23,27^

A model accounting for the fractional site populations (*f*) was used to fit the time-dependent anisotropy ratio function obtained from the experiments. This model is given by
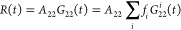
1where *A*_22_ is the unperturbed angular correlation coefficient, and the perturbation factor *G*_22_(*t*) provides specific information about the hyperfine interactions.

For magnetic interactions, *G*_22_(*t*) = 0.2 + 0.4 cos (ω_L_*t*) + 0.4 cos(2ω_L_*t*), where ω_L_ = μ_N_g*B*_hf_/ℏ is the Larmor frequency (note that ν_m_ = ω_L_/2π), μ_N_ is the nuclear magneton and g is the nuclear g-factor. Thus, it is possible to determine the magnetic hyperfine field (*B*_hf_) from the Larmor frequency extracted from measurements of *G*_22_(*t*).^28^

*G*_22_(*t*) = *S*_20_+∑_*n* = 1_^3^*S*_2*n*_(η)cos(ω_*n*_*t*) represents the perturbation function for electric quadrupole interactions. The transition frequencies ω_*n*_ are related to the quadrupole frequency ν_Q_ = (eQV_*zz*_)/*h* through the relationship ω_*n*_ = *g*_*n*_(η)ν_Q_. These transition frequencies, as well as *S*_2*n*_(η) and *g*_*n*_(η) are known to be functions of the asymmetry parameter (η). The asymmetry parameter is defined as η = (*V*_*xx*_ – *V*_*yy*_)/*V*_*zz*_ and is dependent on the diagonal components of the EFG tensor, *V*_*kk*_, under the assumption that |*V*_*xx*_| ≤ |*V*_*yy*_| ≤ |*V*_*zz*_|. Lastly, Q is the electric quadrupole moment of the probe nuclei. For both quadrupole electric and dipole magnetic static interactions, the width of the frequency distribution is given by a Lorentz broadening, which is parametrized by δω exponential factor.

Given the well-known Q of the probe nucleus, the major component (*V*_*zz*_) of the EFG can be calculated through the experimental determination of ν_Q_. Additionally, the measure of η offers information on the configuration of the EFG in the crystallographic site where the probe nuclei are located. The local distortion of the crystallographic site causes a variation in the local charge distribution from axial symmetry, which is measured by the parameter η, which has a range of values from 0 to 1. It is important to emphasize that η provides information on the local symmetry surrounding the probe nucleus.

## Results and Discussion

### Structural Characterization

The diffraction patterns of samples S_1_ and S_2_, as shown in Figure 1, reveal distinct crystal structures. For sample S_1_, the peaks corresponding to the crystalline planes (111), (200), (220), (311) and (222) indicate a cubic crystal structure of the rocksalt (fcc-CoO), with lattice parameters of *a* = *b* = *c* = 4.2500 Å. The mean crystallite size, estimated using the Scherrer equation, is approximately 7 nm. In contrast, sample S_2_ exhibits peaks corresponding to the (1 0 0), (0 0 2), (1 0 1), (1 0 2), (1 1 0), (1 0 3), (2 0 0), (1 1 2), and (2 0 1) planes, characteristic of a closed-packed hexagonal structure of cobalt monoxide (hcp-CoO), also known as Wurtzite. The lattice parameters obtained from the Rietveld refinement are *a* = *b* = 3.2100 and *c* = 5.2400 Å, consistent with the values in the literature.^18^ The mean crystallite size, of S_2_ sample, determined using the Scherrer equation, is approximately 28 nm.

**Figure 2:**
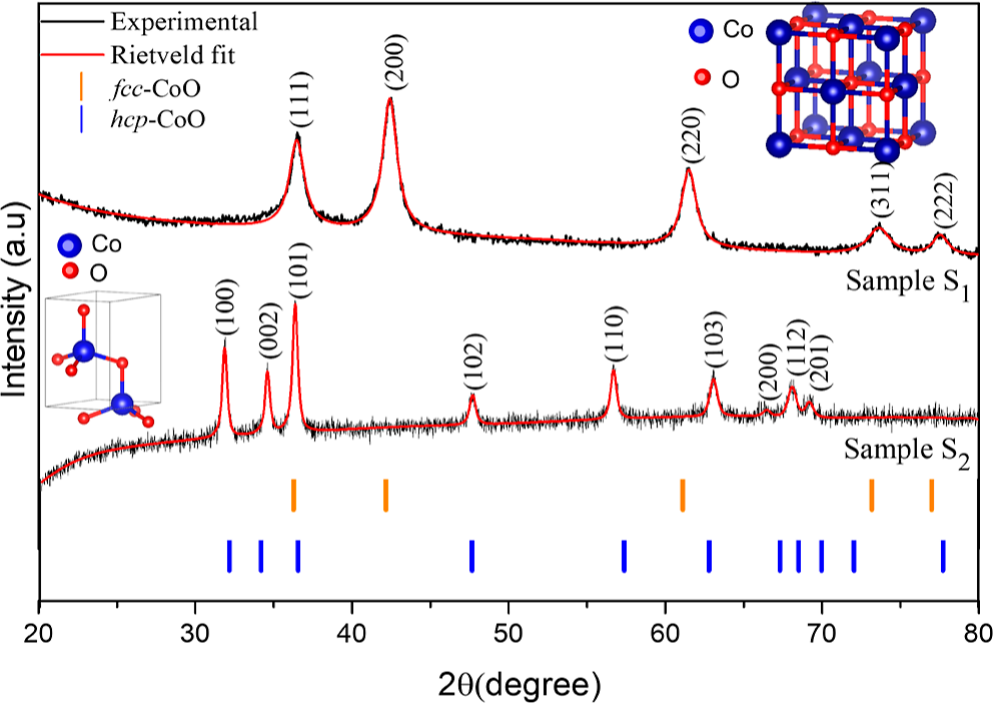
X-ray diffraction patterns of samples S_1_ and S_2_. The fcc-CoO was determined for sample S_1_ whereas the hcp-CoO was obtained for the sample S_2_. The red curve represents the fit obtained using the Rietveld method, indicating the absence of secondary phases in the samples.

In particular, the absence of additional phases, such as Co_3_O_4_ spinel types, was confirmed by Rietveld refinement for both samples, indicating the pure fcc structure for S_1_ and the pure hcp structure for sample S_2_. The X-ray diffraction patterns presented in Figure 2 represent the distinct crystallographic phases observed for S_1_ and S_2_, with the red curve representing the Rietveld refinement.

These results indicate that rigorous control of parameters such as ligand proportion, temperature, and growth time is crucial for obtaining pure phase CoO nanoparticles, as observed in recent studies.^16^ Notably, the hcp-CoO phase, due to its metastable nature, has been frequently reported as challenging to stabilize and is prone to transition to the Co_3_O_4_ phase at temperatures above 200 ^*°*^C.^29^ In applications involving these systems, structural characteristics such as crystal orientation, crystallite size, and surface defects are crucial, especially in relation to their catalytic and magnetic properties. For instance, crystal orientation is essential for utilizing these systems as supports for the oriented growth of other nanostructures, such as CoFe_2_O_4_, and for optimizing gas selectivity in catalytic processes.^14,30,31^ Additionally, factors such as crystallite size and surface defects significantly influence the magnetization properties of CoO nanoparticles.^15,25,32^ On the other hand, from the perspective of nanoparticles, these characteristics are significantly related to the morphology.^19,33^

**Figure 3:**
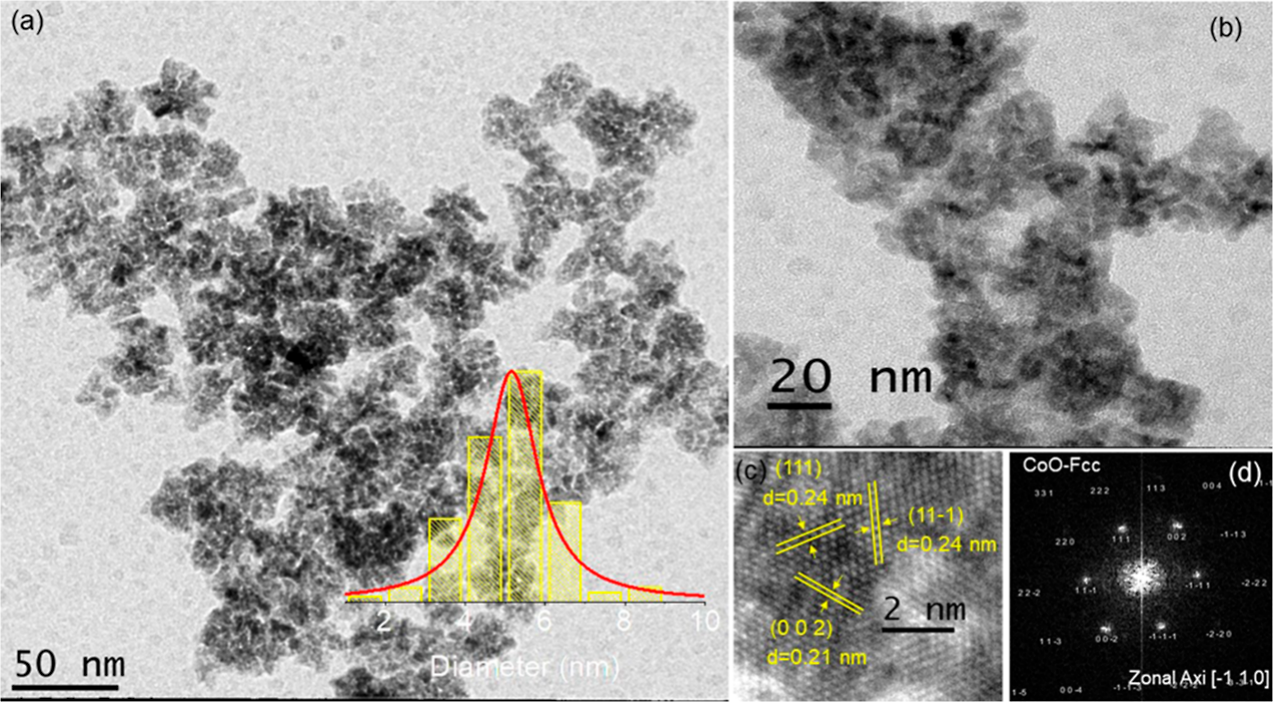
Images of sample S_1_ observed by TEM/HRTEM are presented. (a) The image shows the morphology and size distribution of nanoparticles. (b) Higher magnification micrograph. (c) High-resolution image (HRTEM) displaying the interplanar distance corresponding to the (111) and (002) planes of fcc-CoO. (d) FFT obtained for the high-resolution image, showing the fcc-CoO planes indexed along the [−110] zone axis.

TEM and high-resolution transmission electron microscopy (HRTEM) were used to investigate the morphology of the samples (Figure 3). For sample S_1_ the results indicate the formation of nanoclusters of a size of around 40 nm, resulting from the agglomeration of nanoparticles with an average size of 5 nm and a standard deviation σ = 1.6 nm (Figure 3a,b). The HRTEM image shown in Figure 3c reveals interplanar distances of 0.21 and 0.24 nm corresponding, respectively, to the planes (111) and (002) of fcc-CoO, along the [−110] zone axis as shown in Figure 3d.

**Figure 4:**
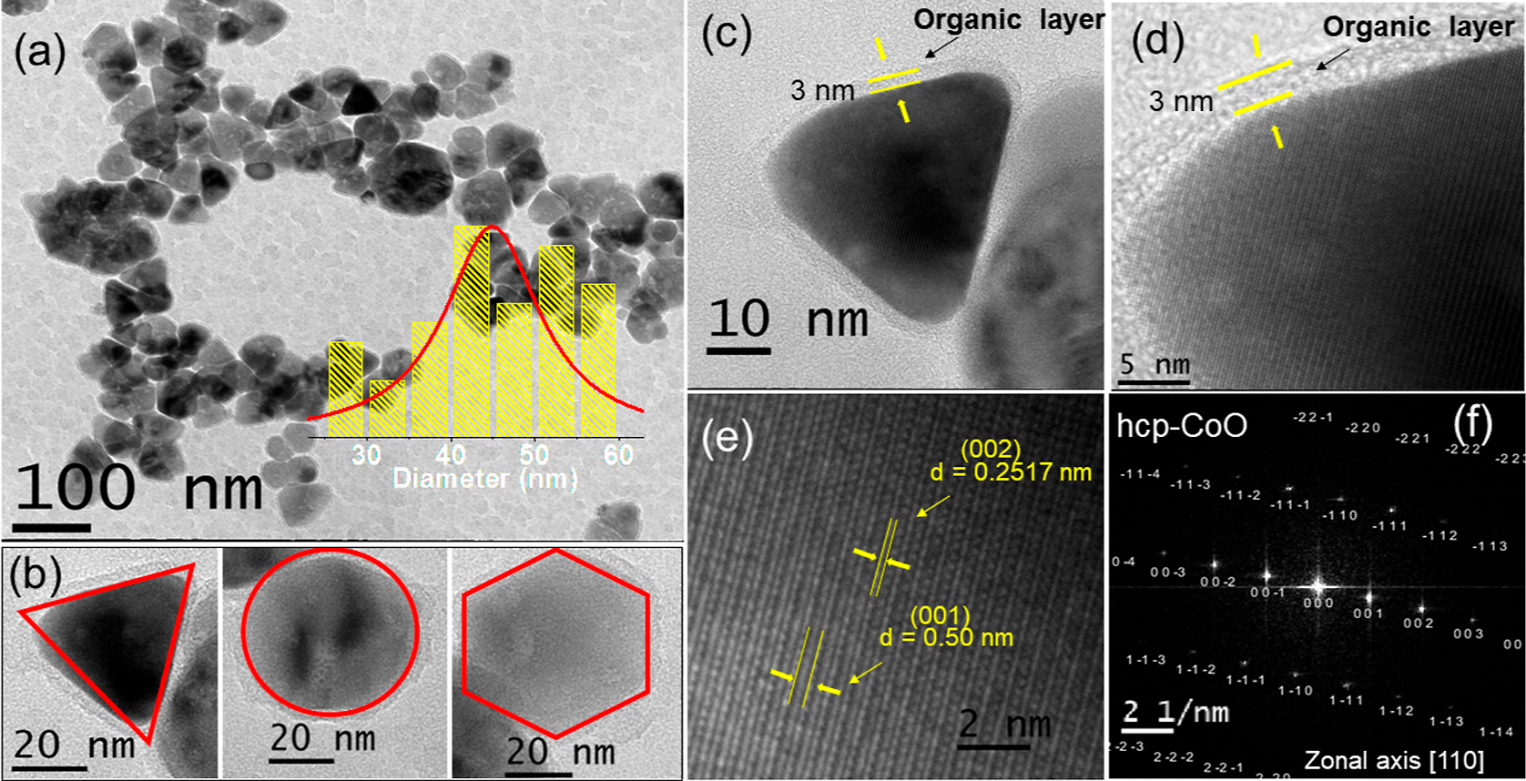
Images of sample S_2_ observed by TEM are presented. (a) Micrograph of the nanoparticles and their size distribution (inset). (b) Nanoparticle morphology. (c,d) Nanoparticles at different magnifications, highlighting the organic coating. (e) HRTEM showing the (001) and (002) planes of hcp-CoO and their respective interplanar distances. (f) FFT obtained for the high-resolution image, showing the hcp-CoO planes indexed along the [110] zone axis.

The images in Figure 4 demonstrate aspects related to size, morphology, and structure for sample S_2_. To investigate the average particle size, a histogram (inset of Figure 4a) was constructed showing nanoparticles with an estimated average size of 43 nm, with a standard deviation σ = 9.3 nm. This result, compared to the average crystallite size of 28 nm obtained by using the Scherrer equation, suggests that a single particle is composed of an assemblage of crystalline domains. Figure 4b illustrates the formation of nanoparticles with different morphologies, including triangular, circular, and hexagonal shapes. These morphological variations may be attributed to the surface (or plane) on which the surfactants are absorbed to stabilize the nanoparticles during the growth stage. The presence of surfactants on the nanoparticle surface is evidenced in Figure 4c, where an organic layer with a thickness of approximately 3 nm can be observed coating the particles. Our observation is in agreement with the research conducted by Jang et al., which suggests that the chemical stabilization of the surface facets is related to OH groups due to the remnant presence of organic material.^34^ The HRTEM image shown in Figure 4e reveals interplanar distances of 0.25 and 0.50 nm attributed to the (002) and (001) planes of hcp-CoO, along the [110] zone axis as indexed in the FFT presented in Figure 4f, which is consistent with the lattice constant of the hcp-CoO structure.

These findings reveal significant differences in the structure, morphology, and size of samples S_1_ (fcc-CoO) and S_2_ (hcp-CoO). These differences indicate that the synthesis conditions such as temperature, chemical concentration, and reaction time strongly influence nanoparticle formation. These synthesis characteristics, which control the size, shape, and structure of nanoparticles, are essential to the kinetics of the nucleation reaction. For example, in fcc-CoO nanoparticles, smaller particles are formed when the nucleation rate exceeds the growth rate. In contrast, bigger nanoparticles, such as hcp-CoO NPs, may develop if the growth rate exceeds the nucleation rate.^35,36^

### Coating Characterization: FTIR

**Figure 5:**
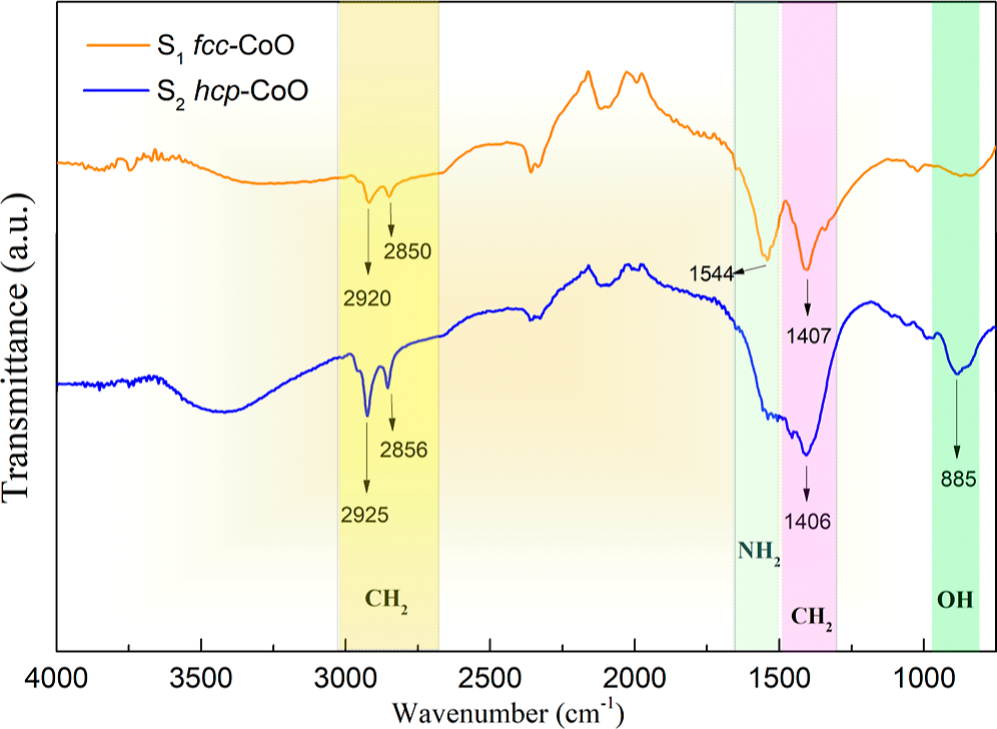
FTIR spectra highlighting the functional groups present in the coating of the nanoparticles, with observed modes NH_2_, CH, CH_2_, and OH. The spectra for sample S_1_ (fcc-CoO) and sample S_2_ (hcp-CoO) are represented by the orange and blue solid lines, respectively.

FTIR measurements are useful for characterizing the coating material of NPs, such as oleylamine and oleic acid for fcc-CoO and oleylamine for hcp-CoO NPs. Figure 5 shows FTIR results for both NPs presenting localized bands at 2850 and 2920 cm^–1^, for sample S_1_, and 2856 and 2925 cm^–1^, for sample S_2_. These bands are characteristic of the fatty acid and fatty amine groups. Oleic acid as well as oleylamine present characteristic modes in these groups, with peaks in the 2851–2853 and 2920–2925 cm^–1^ ranges, which are due to the symmetric and asymmetric stretch modes of the CH_2_.^37,38^ It is difficult to observe other features of the amine group in FTIR measurements because of the superposition of the wide absorption bands of the OH group in the 3200–3500 cm^–1^ range.^39^

The FTIR results for sample S_1_ also reveal a vibration band at 1544 cm^–1^, which is attributed to the distinctive modes of the amine group (scissor mode of NH_2_).^37^ This mode was not observed for sample S_2_ (hcp-CoO) and is associated with oleylamine. The deformation modes of CH in the CH_2_ groups^39^ are correlated with the absorption bands in the 1400–1470 cm^–1^ range. The bands between 869 and 894 cm^–1^ are indicative of deformation outside the OH plane,^40^ and are often present in the carboxylic acids used in the synthesis of the samples. However, the band at 885 cm^–1^ is associated with OH in plane deformation.^40^ Our findings are in agreement with the TEM measurements, which indicate that the stability of the organic–inorganic interface dictates the crystallographic growth orientation of the nanoparticles through the dynamic solvation behavior of the surfactants in solution. This interfacial layer potentially offers an additional design parameter for optimizing the nanoparticles for practical applications.

### Local Characterization

#### Raman Spectroscopy

**Figure 6:**
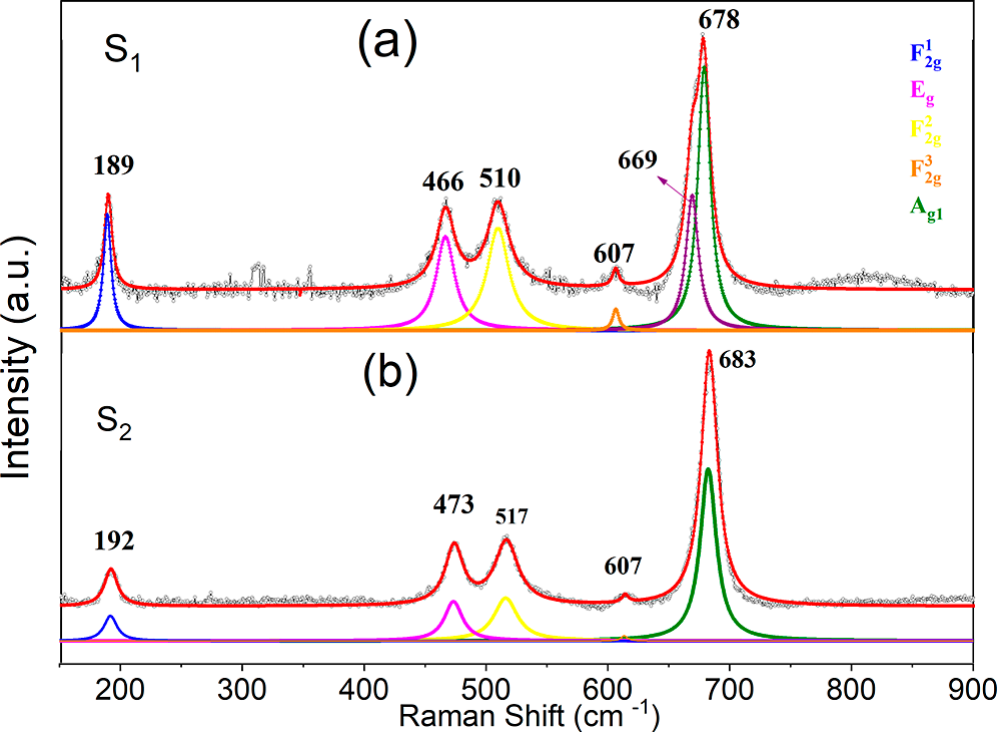
Raman spectra for sample S_1_ (fcc-CoO) at 12 mW (a), and for sample S_2_ (hcp-CoO) at 4 mW (b). The presented spectra show vibrational modes related to the spinel-type Co_3_O_4_ phase, suggesting a phase transition induced by the interaction between the laser and the sample.

At first, both samples were characterized by Raman spectroscopy using a wavelength of 514 nm with laser powers of 12 and 4 mW for samples S_1_ (fcc-CoO) and S_2_ (hcp-CoO), respectively, with the aim of local analysis. The resulting Raman data were treated by the software Fityk that uses the curve-fitting Levenberg*–*Marquardt algorithm. The center and shape of the observed bands for the S_1_ and S_2_ samples were fitted with Lorentzian functions. According to Figure 6a, sample S_1_, which was identified by XRD as fcc-CoO, presents Raman spectra with bands at 192, 473, 517, 607, and 683 cm^–1^. A Raman spectrum with *E*_*g*_, A_1_g, and *T*_2_*g* vibration modes and Raman shifts of about 489, 540, and 690 cm^–1^ should be expected for the fcc-CoO structure.^13^ However, sample S_1_ displays distinct bands of Co_3_O_4_ rather than the typical bands of CoO with fcc structure.

Similarly, sample S_2_ exhibited band centers at positions 189, 466, 510, 607, and 678 cm^–1^. According to theoretical predictions, the hcp-CoO structure with the space group *P*6_3_*mc* should display two characteristic bands corresponding to the vibrational modes A_1_ (LO) and A_1_ (LO + TO) at 565 and 663 cm^–1^, respectively. However, these bands were not observed in the spectrum of S_2_. Instead, the prominent vibrational modes strongly suggest the presence of the cubic spinel structure, belonging to the space group *Fd*_3_*m*, of Co_3_O_4_, which it is not evident in the XRD results. In this structure, Co^2+^ ions occupy the tetrahedral sites (8*a*), while Co^3+^ ions occupy the octahedral sites (16*d*).

Therefore, it can be concluded from the Raman spectrum analysis that the bands observed for samples S_1_ and S_2_ do not correspond to the vibration modes of the fcc-CoO and hcp-CoO phases, respectively. The bands in both spectra are typical of the Co_3_O_4_ phases. These findings suggest that the interaction of CoO nanoparticles with the light beam of the incident laser, which had a power of 12 mW for S_1_ and 4 mW for S_2_, respectively, heated the NPs locally to a temperature high enough to induce a phase transition to the spinel structure of the Co_3_O_4_.

**Figure 7:**
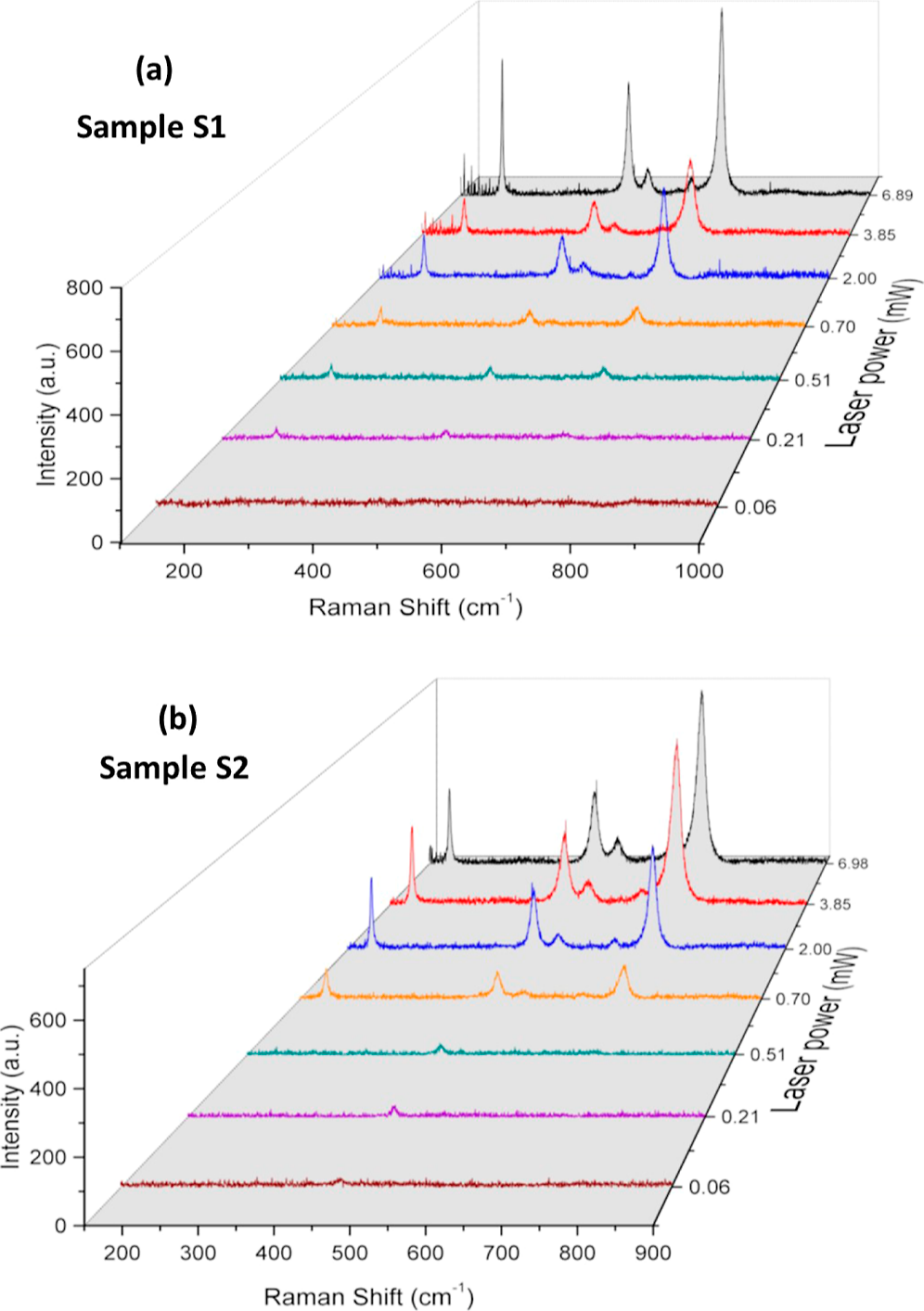
Raman spectra as a function of the laser power to investigate the phase transition by heating induced by the laser incidence on different regions of the CoO nanoparticle samples. (a) Transition from the fcc-CoO to the Co_3_O_4_ structure for sample S_1_. (b) Transition from the wurtzite hcp-CoO to the spinel structure of Co_3_O_4_ for sample S_2_.

To investigate the local annealing induced by the 514 nm laser, variations in the laser power illumination focused at various parts of the sample were carried out. Samples were placed on slide glass and spread out over a 1 cm^2^ area for these measurements. Raman spectra for each sample are shown as a function of laser power in Figure 7. For sample S_1_ (fcc-CoO), the Raman spectrum acquired at the lowest laser power (0.06 mW), as shown in Figure 7a, does not exhibit discernible peaks. This indicates that a higher laser power is necessary to generate a measurable Raman signal. When the laser power is increased to 0.21 mW, bands with centers at 193.49, 482.29, and 684.35 cm^–1^ may be observed. The values of 484 and 691 cm^–1^, reported for the cubic phase of fcc-CoO, attributed to the vibration modes *E*_*g*_ and A_1_*g*^13,17,41^ are very similar to the two later values. In contrast, a shift in the band center positions is observed for laser powers greater than 0.70 mW. These values, which are now at 519.34 and 615.41 cm^–1^, suggest a change from the fcc-CoO structure to the spinel structure of Co_3_O_4_, indicating the presence of new Raman active modes. The Raman spectrum of Co_3_O_4_ is evident at 6.89 mW of laser power.

Results of the laser power variation for sample S_2_ are shown in Figure 7b. The band centers are located at 448.01, 447.82, and 446.40 cm^–1^, respectively, for laser powers of 0.06, 0.20, and 0.51 mW. In the hexagonal close-packed hcp-CoO crystal structure, the *E*_2_ phonon mode is observed alongside other vibrational modes. These vibrational modes correspond to band centers located at 447, 565, and 663 cm^–1^ as previously reported.^42,43^ Consequently, the characteristic A_1_ vibrational mode of hcp-CoO is observed in the Raman spectra acquired at laser power below 0.50 mW. The Raman spectra exhibit a shift when the laser power is increased to 0.70 mW or higher above. These spectra now show band centers at 191.83, 471.75, 511.57, 610.81, and 679.24 cm^–1^, suggesting a full transition to the Co_3_O_4_ phase.

Raman spectroscopy measurements as a function of laser power suggest a local phase transformation in both samples S_1_ and S_2_ to the Co_3_O_4_ structure, with nanoparticles localized in regions exposed to laser irradiation. This phenomenon is triggered by the highly concentrated energy of the laser beam, which raises the temperature of nanoparticles, provoking a change in their crystalline structure. This process is usually referred to as “laser-induced phase transition” and has diverse applications, including nanofabrication and the study of nanoparticle properties.

It is crucial to emphasize that the laser wavelength, its power density, and the irradiation time, along with the intrinsic properties of the nanoparticles, are critical factors governing the induction of the desired phase. This approach offers significant advantages for the controlled modulation of nanoparticle properties, enabling the emergence of novel phenomena at the nanometric scale.

#### Perturbed Angular Correlations

**Figure 8:**
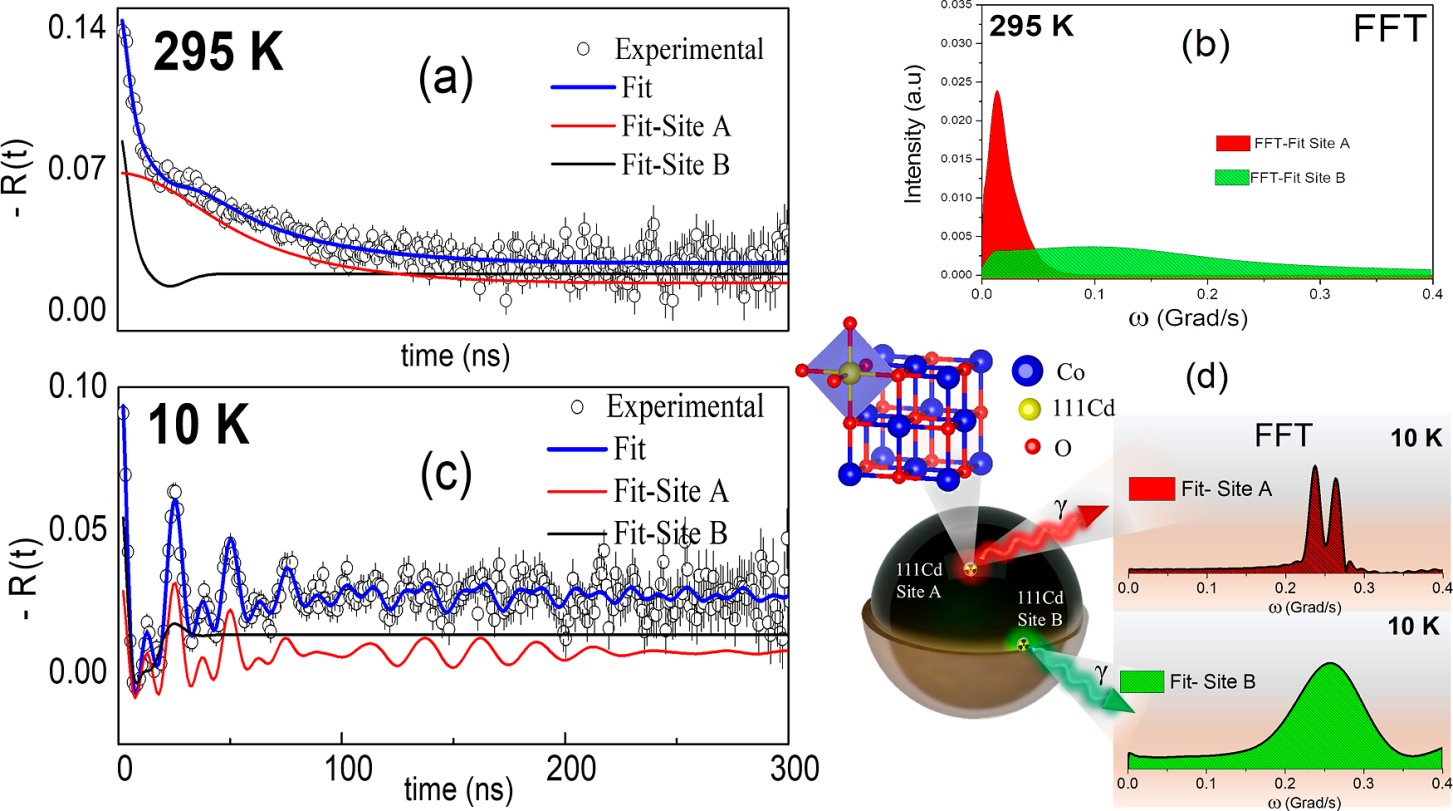
PAC measurements for S_1_. (a) Time-differential anisotropy ratio function *R*(*t*) and (b) the fast Fourier transform (FFT) at room temperature fitted with a two-site model: site A (red) with a pure electric interaction and site B (green) with a broad distributed electric interaction. (c) *R*(*t*) function and (d) its respective fast Fourier transform (FFT) at 10K fitted with a two-site model considering combined electric plus magnetic interactions.

PAC measurements on fcc-CoO (S_1_) and hcp-CoO (S_2_) were carried out using ^111^In(^111^Cd) as probe nucleus at room temperature (RT) and at 10 K, respectively. For S_1_, the time-dependent anisotropy ratio function, *R*(*t*), and their respective fast Fourier transforms, resulting from PAC measurements, are represented in Figure 8. The hyperfine parameters used to fit the *R*(*t*) spectra in Figure 8 are recorded in Table 1, considering a two-site fitting model, namely, site A and site B.

**Table 1:** Hyperfine Interaction Parameters of Sample S_1_ Nanoparticles using a Two-Site model–Site a in the NPs’ Core and Site B in Their Outer layer–at Room Temperature (295 K) and 10 K

site	*T*(*K*)	*f*(%)	ν_Q_(MHz)	ν_M_(MHz)	η	δ(%)
A	295	40.0 ± 1.0	11.0 ± 0.5	0.0 ± 0.0	0.0	52.0 ± 7.0
B	295	60.0 ± 1.0	64.0 ± 7.0	0.0 ± 0.0	0.7	83.0 ± 9.0
A	10	37.0 ± 1.0	8.0 ± 0.5	40.2 ± 0.1	0.0	1.0 ± 0.1
B	10	63.0 ± 1.0	17.0 ± 0.5	42.4 ± 0.7	0.0	15.0 ± 2.0

At RT, a symmetric (η = 0) quadrupole interaction was found to represent site A that is characterized by a low quadrupole frequency ν_QA_ = 11 MHz with a very broad distribution δ_A_ = 52% and a site fraction of *f*_A_ = 40%. This low quadrupolar interaction corresponds to the probe nucleus substituting a cation position in the crystalline structure (see the continuous red line fit). Site B, on the other hand, presents a higher fractional population (*f*_B_ = 60%), characterized by a wide frequency distribution (δ_A_ = 83%) and a ν_QB_ ∼ 64 MHz (see the continuous green line fit), which was attributed to the probe nuclei occupying the outer layer of nanoparticles rich in residual organic material.

For the PAC measurements on S_1_ at 10 K, the *R*(*t*) spectrum shown in Figure 8c, exhibits characteristic oscillations due to the transferred magnetic hyperfine field (*B*_hf_) interaction with a well-defined frequency (δ_A_ = 1%), as seen in the FFT spectrum (see Figure 8d). The fitting was performed with a two-site fraction model, where site A has a population *f*_A_ = 37%, with ν_QA_ ∼ 8 MHz and Larmor frequency ν_M_ = 40.2 MHz, equivalent to *B*_hf_ = 17 T. This site shows a low-frequency distribution attributed to the substitutional position of the probe nucleus. Site B has *f*_B_ = 63% with δ_A_ = 15%. This fractional population has been assigned to the probe nuclei in the outer layer of the particles. At this site, ν_QB_ = 17 MHz and ν_M_ = 42.4 MHz, which is equivalent to *B*_hf_ = 18 T. The data are consistent with the systematic work published by Santos et al. on CoO, bulk, and NPs.^25^ At the external regions of the NPs, low symmetry and crystallinity are attributed to the amorphous formation of the organic material coating the NPs. It is important to mention that coating or encapsulation plays an important role in the stability and functionality of nanoparticles.

**Figure 9:**
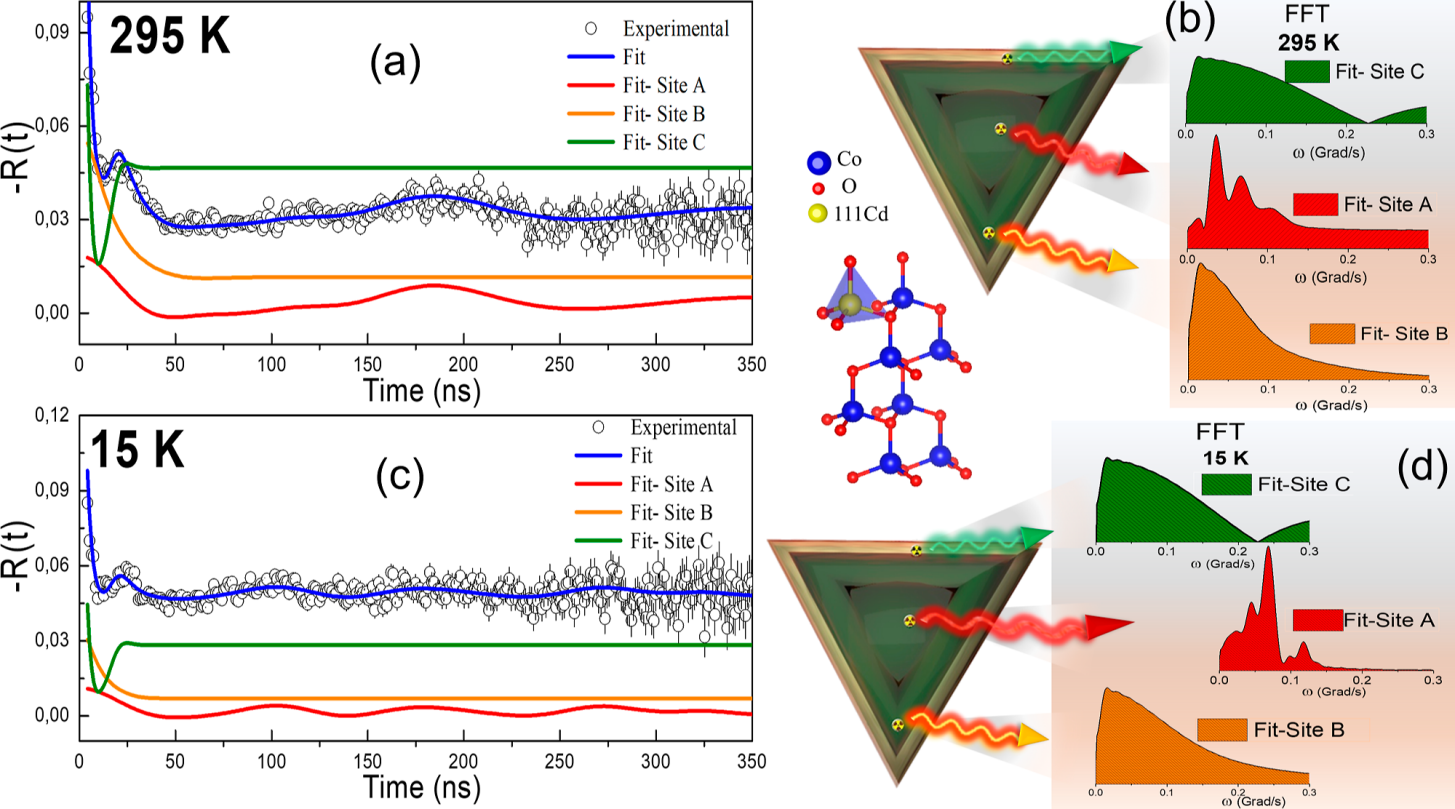
PAC measurements for S_2_ (a) time-differential anisotropy ratio function *R*(*t*), and (b) the fast Fourier transform (FFT) at room temperature fitted with a three-site model considering pure electric quadrupole interactions: site A (red), and site B (orange) and site C (green), both with broad frequency distributions. (c) Time-differential anisotropy ratio function *R*(*t*) and (d) its corresponding fast Fourier transform (FFT) at 15 K fitted with a three-site model considering for site A a combined electric plus magnetic interactions, and for sites B and C pure electric quadrupole interactions.

The time-dependent anisotropy ratio function *R*(*t*) of sample S_2_ at room temperature displayed pronounced damping. The hyperfine parameters used for the fit of the *R*(*t*) spectra in Figure 9 were recorded in Table 2, considering a model of three-site fitting, site A, site B and site C, which reveals valuable information about the local structure (see Figure 9a). Site A (red line) can be interpreted as the occupation of the probe nucleus, ^111^In(^111^Cd), in crystalline substitutional sites in pyramidal or hexagonal sample nanoparticles, as it showed a δ = 10% (Figure 9b). This high crystallinity phase was identified in the internal regions of the NPs, as shown in the TEM images in Figure 4e. The electric quadrupolar frequency extracted from the *R*(*t*) spectrum was ν_QA_ ∼33 MHz, and this value matches the value reported in Pereira et al. for the impurities ^111^ Cd located at defect-free cation sites in the codoped ZnO structure (whose value is ν_QA_ ∼33 MHz).^44^ In addition, taking into account the Co solubility threshold in ZnO, a stable physical situation occurs in this region: with an increase in Co doping concentration in the ZnO host matrix, the system exhibits a solution-like behavior, favoring the formation of diluted clusters and the consolidation of metastable phases such as a ZnO–CoO. In other words, the CoO clusters in Pereira et al., have the same space group (*P*6_3_*mc*) and point group (6*mm*) of the hcp-CoO sample S_2_ nanoparticles (see Figure 9a), justifying the similar hyperfine parameters obtained from both studies. This is seen more clearly in the correspondent band structure calculations,^44^ where the Cd and Co impurity bands are fully hybridized with the band characteristic of the ZnO host matrix (bands coexisting at the same energy level).

**Table 2:** Hyperfine Interaction Parameters of Sample S_2_ Nanoparticles at Room Temperature (295 K) and 15 K using a Three-Site Model: Site a Inside the NPs, Site B Surface the NPs, and Site C Outside Region

site	*T*(*K*)	*f*(%)	ν_Q_(MHz)	ν_M_(MHz)	η	δ(%)
A	295	17.0 ± 1.0	33.0 ± 0.4	0.0	0.3	10.0 ± 1.2
B	295	17.0 ± 1.0	29.0 ± 0.6	0.0	0.0	33.0 ± 4.5
C	295	66.0 ± 1.0	207.0 ± 9.51	0.0	0.3	60.0 ± 7.0
A	15	15.0 ± 1.0	33.0 ± 0.7	2.5 ± 0.2	0.3	5.0 ± 0.2
B	15	15.0 ± 1.0	29.0 ± 0.6	0.0	0.3	40.0 ± 8.0
C	15	70.0 ± 1.0	207.0 ± 0.8	0.0	0.5	31.0 ± 3.0

At 15 K, the sample S_2_, hcp-CoO nanoparticles, exhibit relatively low magnetic interaction associated with weak magnetic coupling. A combined interaction model (electric quadrupolar plus magnetic dipolar interactions) was used to fit site A at 15 K, which produced a magnetic Larmor frequency, ν_M_ = 2.5 MHz, equivalent to a transferred hyperfine field of *B*_hf_ = 0.86 T, significantly lower than *B*_hf_ of the fcc-CoO phase, which was *B*_hf_ ∼ 17 T at 10 K. This aspect (low hyperfine field), reinforces the hypothesis of weak magnetic coupling in this system, as suggested by several authors through electron spin resonance (ESR) spectroscopy measurements.^17,45^

Site B (orange line) has been identified as probe nuclei located in a region close to the surface of the NPs, characterized by bond breaks and low crystallinity. The low crystallinity at RT was confirmed by the observation of ν_QB_ = 29.0(1) MHz with a broad frequency distribution (δ = 33%) at this site. Furthermore, no transferred hyperfine field was seen at low temperatures, such as 15 K, because of the weak magnetic coupling in the hcp-CoO system, which is susceptible to symmetry breaks and defects that are typical of the surface region in these NPs.

At site C (green line), there is a high fractional population (*f*_C_ = 66%) with a high electrical frequency value (ν_QC_ ≈207 MHz), with η = 0.3, and δ = 60%. This broad frequency distribution characterizes probe nuclei in the outer region of the nanoparticles, which are made up of residual amorphous organic material. The presence of organic material surrounding the nanoparticles was confirmed by FTIR measurements and TEM images, as shown in Figures 5 and 4c,d, respectively.

Finally, the analysis of substitutional sites in samples S_1_ and S_2_ at low-temperature measurements revealed a weak transferred hyperfine field (*B*_hf_) for ^111^In(^111^Cd) in the hcp-CoO crystal lattice (S_2_), which was approximately *B*_hf_ = 1 T. This value is significantly lower than that observed in the fcc-CoO phase (S_1_), which was around 17 T. The superexchange interaction, in which the spin density is transferred from the paramagnetic Co ions to the diamagnetic Cd ions via O ions (through the Co–O–Cd bonds), is the cause of the weakening of the hyperfine magnetic field at the Cd probes in hcp-CoO. This transfer takes place through the spin polarization of the closed s shells of Cd by magnetic neighbors through the overlap of the oxygen p orbital, transferring unpaired spin density into the outermost Cd 5ıs orbital.^46^

Since orbitals with rotational symmetry around the bond axis (*e*_g_ orbitals: *d*_*z*_^2^ and ) transfer maximum spin density, these should be considered in the first place. If *e*_g_ orbitals are empty, the *t*_2_*g* orbitals (*d*_*xy*_, *d*_*yz*_, *d*_*zx*_), which have relatively small overlap with the orbitals of nearby oxygen ions, transfer less spin density to Cd orbitals via Co–O–Cd exchange bonds. As a consequence, the supertransferred magnetic hyperfine field at ^111^Cd nuclei would be smaller for Co–O–Cd bond angles closer to 90° than those closer to 180°.

Therefore, the origin of the big difference in *B*_hf_ is essentially due to the different geometries in both systems. The hcp-CoO structure has a hexagonal geometry, with a complex magnetic structure that induces spin frustration, weakening the superexchange mechanism through Co^2+^–O^2–^–Co^2+^ bonds due to the characteristic angle of 110°, also known as two-dimensional frustration.^17,47−49^ However, the superexchange interaction is stronger in the rock-salt structure, featuring an antiferromagnetic type II (AFM-II) magnetic structure, favored by linear superexchange between Co^2+^–O^2–^–Co^2+^ bonds with an angle of 180°.^50,51^ Additionally, it is also suggested that the magnetism in hcp-CoO is possibly dependent on the morphology and type of defects in its structure (e.g., oxygen vacancies, stacking faults, among others, provided by synthesis procedure), which may contribute to different manifestations of magnetic behaviors.

## Conclusions

Samples of CoO nanoparticles were synthesized with the thermal decomposition method using two different ligand pairs: one sample with the oleylamine/oleic acid ligand pair, and the other with only oleylamine. Data analysis of X-ray patterns revealed characteristic patterns of the unique phases for each sample after synthesis, rock-salt cubic fcc phase with an average size of 5 nm, and wurtzite hexagonal hcp phase with a size of 43 nm, respectively.

Raman spectra analysis indicated similarities in bands for both samples, suggesting a localized phase transformation process induced by laser-power density, resulting in the formation of the Co_3_O_4_. The measurements reveal that the surfaces of both fcc-CoO and hcp-CoO NPs are susceptible and unstable under incident light power, which may influence the properties of the NPs triggering a local crystalline phase transition to Co_3_O_4_ as observed.

PAC measurements for doped samples, with probe nuclei ^111^In(^111^Cd), during the synthesis process, revealed weaker transferred hyperfine fields in the hcp-CoO phase (*B*_hf_ = 1 T) compared to the fcc-CoO phase (*B*_hf_ = 17 T), indicating that superexchange interactions are strongly influenced by the angle 110° for hcp-CoO and an angle 180° for fcc-CoO due to the system geometries. Both Raman and PAC measurements show low crystallinity on the surfaces of the NPs, as revealed by the low intensity of the Raman bands at low powers and the high damping (δ) of the *R*(*t*) spectra. The complementary techniques provide valuable insights into the crystalline and magnetic properties of CoO polymorphs.
